# Ammonium 2-(2,4-dichloro­phen­oxy)acetate hemihydrate

**DOI:** 10.1107/S1600536809026919

**Published:** 2009-07-18

**Authors:** Hui-Lian Liu, Shu-Hua Guo, Yun-Ying Li, Fang-Fang Jian

**Affiliations:** a Microscale Science Institute, Biology Department, Weifang University, Weifang 261061, People’s Republic of China; b The 7th Middle School, Weifang 261061, People’s Republic of China; c Microscale Science Institute, Weifang University, Weifang 261061, People’s Republic of China

## Abstract

The title compound, NH_4_^+^·C_8_H_7_Cl_2_O_6_^−^·0.5H_2_O, was prepared by the reaction of 2-(2,4-dichloro­phen­oxy)­acetic acid and ammonia in water at 367 K. The mol­ecular structure and packing are stabilized by N—H⋯O and O—H⋯O inter­molecular hydrogen-bond inter­actions.

## Related literature

For the biological activity of 2-(2,4-dichloro­phen­oxy)acetic acid, see: Lv *et al.* (1998 ▶). Due to their versatile bonding modes with metal ions, they have also been used in the synthesis of mononuclear monomeric (Gao *et al.*, 2004*a* ▶; Psomas *et al.*, 2000 ▶) and polymeric complexes (Liu *et al.*, 2004 ▶; Gao *et al.*, 2004*b* ▶, 2005 ▶).
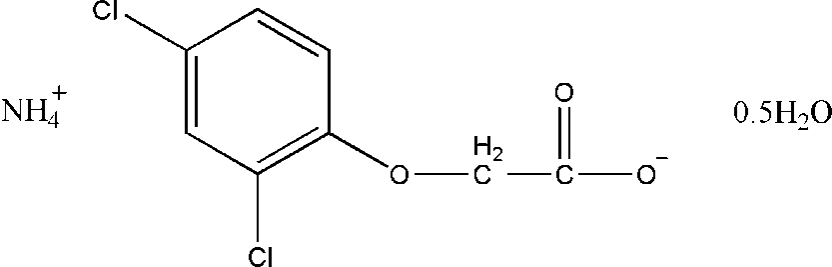


## Experimental

### 

#### Crystal data

NH_4_^+^·C_8_H_5_Cl_2_O_3_^−^·0.5H_2_O
*M* *_r_* = 247.07
Monoclinic, 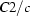

*a* = 37.738 (8) Å
*b* = 4.3889 (9) Å
*c* = 12.900 (3) Å
β = 103.83 (3)°
*V* = 2074.7 (8) Å^3^
*Z* = 8
Mo *K*α radiation
μ = 0.61 mm^−1^
*T* = 293 K
0.15 × 0.12 × 0.10 mm

#### Data collection

Bruker SMART CCD area-detector diffractometer
Absorption correction: none
9447 measured reflections
2385 independent reflections
2216 reflections with *I* > 2σ(*I*)
*R*_int_ = 0.026

#### Refinement

*R*[*F* ^2^ > 2σ(*F* ^2^)] = 0.023
*wR*(*F* ^2^) = 0.060
*S* = 1.07
2385 reflections
137 parameters
90 restraints
H-atom parameters constrained
Δρ_max_ = 0.38 e Å^−3^
Δρ_min_ = −0.19 e Å^−3^

### 

Data collection: *SMART* (Bruker, 1997 ▶); cell refinement: *SAINT* (Bruker, 1997 ▶); data reduction: *SAINT*; program(s) used to solve structure: *SHELXS97* (Sheldrick, 2008 ▶); program(s) used to refine structure: *SHELXL97* (Sheldrick, 2008 ▶); molecular graphics: *SHELXTL* (Sheldrick, 2008 ▶); software used to prepare material for publication: *SHELXTL*.

## Supplementary Material

Crystal structure: contains datablocks global, I. DOI: 10.1107/S1600536809026919/at2826sup1.cif

Structure factors: contains datablocks I. DOI: 10.1107/S1600536809026919/at2826Isup2.hkl

Additional supplementary materials:  crystallographic information; 3D view; checkCIF report

## Figures and Tables

**Table 1:** Hydrogen-bond geometry (Å, °)

*D*—H⋯*A*	*D*—H	H⋯*A*	*D*⋯*A*	*D*—H⋯*A*
O1*W*—H1⋯O2^i^	0.85	1.97	3.2969 (15)	170
N1—H1*A*⋯O2^i^	0.84	2.07	2.8908 (14)	168
N1—H1*B*⋯O3^ii^	0.85	2.02	2.8578 (13)	168
N1—H1*C*⋯O3^iii^	0.88	2.09	2.9310 (15)	161

Symmetry codes: (i) 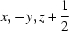; (ii) 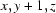; (iii) 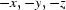.

**Fig. 1.**
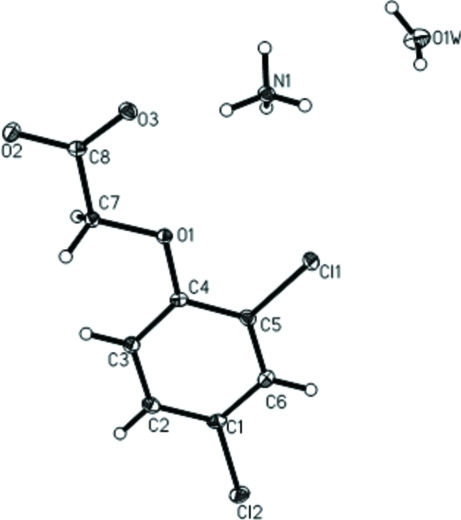
The molecular structure of the title compound showing 30% probability displacement ellipsoids and the atom-numbering scheme.

**Fig. 2.**
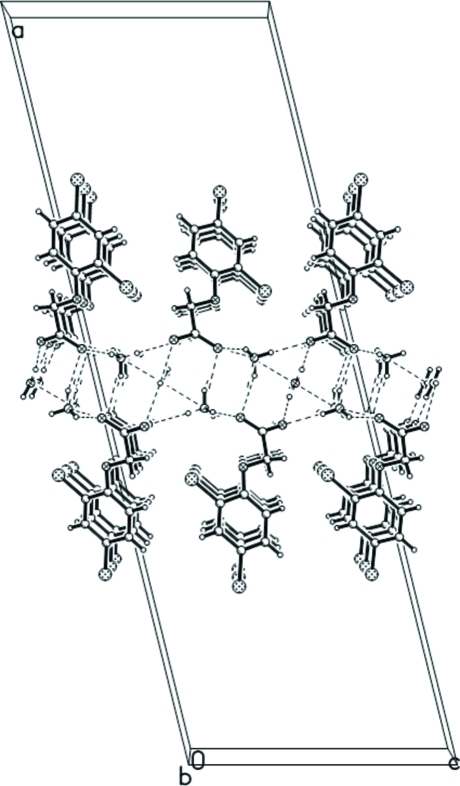
The crystal packing of (I), viewed along b axis. Hydrogen bonds are indicated by dashed lines.

## supplementary crystallographic information

### Comment

2-(2,4-Dichlorophenoxy)acetic acid is one of the important biologically active compounds that have been commonly used in herbicides and plant growth substances (Lv *et al.*, 1998). Due to their versatile bonding modes with metal ions, they have also been used in the synthesis of mononuclear monomeric (Gao *et al.*, 2004*a*; Psomas *et al.*, 2000) and polymeric complexes (Liu *et al.*, 2004; Gao *et al.*, 2004*b*, 2005). We synthesized the title compound, (I), and report here its crystal structure.

In the crystal structure of (I) (Fig. 1), all the non-H atoms of 2-(2,4-dichlorophenoxy)acetic acid are in the same plane, with the maximum deviation being 0.146Å for atom O3.

### Experimental

A mixture of 2-(2,4-dichlorophenoxy)acetic acid (4.42 g, 0.02 mol) and ammonia (1.0 ml, 0.02 mol) was stirred with water (50 ml) at 367 K for 2 h. Single crystals suitable for X-ray measurements were obtained by recrystallization from acetone and ethanol (1:1) at room temperature.

### Refinement

The H atoms of the water molecule were found from a difference Fourier map and refined freely. The remaining H atoms were fixed geometrically and allowed to ride on their attached atoms, with C—H and N—H distances of 0.93–0.96 and 0.86Å, respectively, and with *U*_iso_(H)=1.2–1.5*U*_eq_(C,N).

### Crystal data

**Table:**

NH_4_^+^·C_8_H_5_Cl_2_O_3_^−^·0.5H_2_O	*F*(000) = 1016
*M**_r_* = 247.07	*D*_x_ = 1.582 Mg m^−^^3^
Monoclinic, *C*2/*c*	Mo *K*α radiation, λ = 0.71073 Å
Hall symbol: -C 2yc	Cell parameters from 2216 reflections
*a* = 37.738 (8) Å	θ = 3.2–27.5°
*b* = 4.3889 (9) Å	µ = 0.61 mm^−^^1^
*c* = 12.900 (3) Å	*T* = 293 K
β = 103.83 (3)°	Bar, colourless
*V* = 2074.7 (8) Å^3^	0.15 × 0.12 × 0.10 mm
*Z* = 8	

### Data collection

**Table:**

Bruker SMART CCD area-detector diffractometer	2216 reflections with *I* > 2σ(*I*)
Radiation source: fine-focus sealed tube	*R*_int_ = 0.026
graphite	θ_max_ = 27.5°, θ_min_ = 3.2°
φ and ω scans	*h* = −48→48
9447 measured reflections	*k* = −5→5
2385 independent reflections	*l* = −16→16

### Refinement

**Table:**

Refinement on *F*^2^	Primary atom site location: structure-invariant direct methods
Least-squares matrix: full	Secondary atom site location: difference Fourier map
*R*[*F*^2^ > 2σ(*F*^2^)] = 0.023	Hydrogen site location: inferred from neighbouring sites
*wR*(*F*^2^) = 0.060	H-atom parameters constrained
*S* = 1.07	*w* = 1/[σ^2^(*F*_o_^2^) + (0.0294*P*)^2^ + 1.6158*P*] where *P* = (*F*_o_^2^ + 2*F*_c_^2^)/3
2385 reflections	(Δ/σ)_max_ < 0.001
137 parameters	Δρ_max_ = 0.38 e Å^−^^3^
90 restraints	Δρ_min_ = −0.19 e Å^−^^3^

### Special details

**Table:**

Geometry. All e.s.d.'s (except the e.s.d. in the dihedral angle between two l.s. planes) are estimated using the full covariance matrix. The cell e.s.d.'s are taken into account individually in the estimation of e.s.d.'s in distances, angles and torsion angles; correlations between e.s.d.'s in cell parameters are only used when they are defined by crystal symmetry. An approximate (isotropic) treatment of cell e.s.d.'s is used for estimating e.s.d.'s involving l.s. planes.
Refinement. Refinement of *F*^2^ against ALL reflections. The weighted *R*-factor *wR* and goodness of fit *S* are based on *F*^2^, conventional *R*-factors *R* are based on *F*, with *F* set to zero for negative *F*^2^. The threshold expression of *F*^2^ > σ(*F*^2^) is used only for calculating *R*-factors(gt) *etc*. and is not relevant to the choice of reflections for refinement. *R*-factors based on *F*^2^ are statistically about twice as large as those based on *F*, and *R*- factors based on ALL data will be even larger.

### Fractional atomic coordinates and isotropic or equivalent isotropic displacement parameters (Å^2^)

**Table:**

	*x*	*y*	*z*	*U*_iso_*/*U*_eq_	
Cl2	0.252351 (7)	0.68291 (7)	0.14126 (2)	0.01935 (8)	
Cl1	0.120306 (7)	0.46470 (7)	0.21755 (2)	0.01934 (8)	
O3	0.04445 (2)	−0.21277 (17)	−0.01018 (6)	0.01408 (16)	
O2	0.05453 (2)	−0.41964 (18)	−0.15912 (6)	0.01545 (16)	
O1	0.10714 (2)	0.10909 (18)	0.02392 (6)	0.01371 (16)	
C4	0.14091 (3)	0.2393 (2)	0.04524 (8)	0.0119 (2)	
C6	0.18475 (3)	0.5588 (2)	0.16711 (8)	0.0146 (2)	
H6A	0.1910	0.6774	0.2285	0.018*	
C7	0.09822 (3)	−0.0753 (2)	−0.07012 (8)	0.0122 (2)	
H7A	0.1178	−0.2195	−0.0687	0.015*	
H7B	0.0961	0.0534	−0.1324	0.015*	
C3	0.16595 (3)	0.2075 (2)	−0.01809 (9)	0.0141 (2)	
H3A	0.1598	0.0924	−0.0803	0.017*	
C8	0.06265 (3)	−0.2485 (2)	−0.07926 (8)	0.0113 (2)	
C5	0.15090 (3)	0.4193 (2)	0.13763 (8)	0.0130 (2)	
C2	0.20009 (3)	0.3460 (3)	0.01088 (9)	0.0152 (2)	
H2A	0.2166	0.3227	−0.0316	0.018*	
C1	0.20925 (3)	0.5183 (2)	0.10313 (9)	0.0142 (2)	
N1	0.03308 (2)	0.2772 (2)	0.11575 (7)	0.01333 (18)	
O1W	0.0000	−0.1574 (3)	0.2500	0.0237 (3)	
H1A	0.0399	0.2902	0.1824	0.024 (4)*	
H1B	0.0378	0.4414	0.0872	0.024 (4)*	
H1WA	−0.0158	−0.2797	0.2154	0.044 (5)*	
H1C	0.0093	0.2577	0.0997	0.029 (4)*	
H1D	0.0423	0.1190	0.0853	0.029 (4)*	

### Atomic displacement parameters (Å^2^)

**Table:**

	*U*^11^	*U*^22^	*U*^33^	*U*^12^	*U*^13^	*U*^23^
Cl2	0.01261 (13)	0.02457 (15)	0.02073 (14)	−0.00724 (10)	0.00367 (10)	−0.00251 (10)
Cl1	0.01548 (13)	0.02906 (16)	0.01553 (13)	−0.00433 (11)	0.00777 (10)	−0.00675 (10)
O3	0.0133 (3)	0.0140 (4)	0.0165 (4)	−0.0013 (3)	0.0066 (3)	−0.0002 (3)
O2	0.0164 (4)	0.0157 (4)	0.0137 (4)	−0.0026 (3)	0.0024 (3)	−0.0021 (3)
O1	0.0108 (3)	0.0175 (4)	0.0137 (4)	−0.0038 (3)	0.0047 (3)	−0.0044 (3)
C4	0.0099 (4)	0.0119 (5)	0.0134 (5)	−0.0002 (4)	0.0019 (4)	0.0022 (4)
C6	0.0150 (5)	0.0155 (5)	0.0125 (5)	−0.0014 (4)	0.0013 (4)	−0.0006 (4)
C7	0.0119 (5)	0.0132 (5)	0.0121 (5)	−0.0014 (4)	0.0042 (4)	−0.0020 (4)
C3	0.0137 (5)	0.0150 (5)	0.0140 (5)	−0.0012 (4)	0.0040 (4)	−0.0013 (4)
C8	0.0101 (4)	0.0101 (4)	0.0130 (5)	0.0014 (4)	0.0016 (4)	0.0033 (4)
C5	0.0125 (5)	0.0154 (5)	0.0121 (5)	0.0007 (4)	0.0048 (4)	0.0012 (4)
C2	0.0130 (5)	0.0171 (5)	0.0170 (5)	−0.0006 (4)	0.0064 (4)	0.0010 (4)
C1	0.0102 (5)	0.0151 (5)	0.0167 (5)	−0.0028 (4)	0.0019 (4)	0.0021 (4)
N1	0.0131 (4)	0.0134 (4)	0.0143 (4)	−0.0009 (3)	0.0050 (3)	0.0001 (3)
O1W	0.0199 (6)	0.0128 (5)	0.0358 (7)	0.000	0.0014 (5)	0.000

### Geometric parameters (Å, °)

**Table:**

Cl2—C1	1.7400 (11)	C7—H7A	0.9700
Cl1—C5	1.7336 (12)	C7—H7B	0.9700
O3—C8	1.2584 (13)	C3—C2	1.3924 (15)
O2—C8	1.2523 (13)	C3—H3A	0.9300
O1—C4	1.3635 (13)	C2—C1	1.3824 (16)
O1—C7	1.4298 (12)	C2—H2A	0.9300
C4—C3	1.3962 (15)	N1—H1A	0.8385
C4—C5	1.4042 (15)	N1—H1B	0.8474
C6—C5	1.3851 (15)	N1—H1C	0.8757
C6—C1	1.3901 (16)	N1—H1D	0.9069
C6—H6A	0.9300	O1W—H1WA	0.8458
C7—C8	1.5225 (14)		
			
C4—O1—C7	115.30 (8)	O2—C8—C7	113.65 (9)
O1—C4—C3	124.81 (10)	O3—C8—C7	120.22 (9)
O1—C4—C5	117.10 (9)	C6—C5—C4	121.65 (10)
C3—C4—C5	118.09 (10)	C6—C5—Cl1	119.18 (8)
C5—C6—C1	118.74 (10)	C4—C5—Cl1	119.17 (8)
C5—C6—H6A	120.6	C1—C2—C3	119.65 (10)
C1—C6—H6A	120.6	C1—C2—H2A	120.2
O1—C7—C8	111.88 (9)	C3—C2—H2A	120.2
O1—C7—H7A	109.2	C2—C1—C6	121.08 (10)
C8—C7—H7A	109.2	C2—C1—Cl2	119.67 (9)
O1—C7—H7B	109.2	C6—C1—Cl2	119.24 (9)
C8—C7—H7B	109.2	H1A—N1—H1B	110.0
H7A—C7—H7B	107.9	H1A—N1—H1C	107.2
C2—C3—C4	120.77 (10)	H1B—N1—H1C	106.9
C2—C3—H3A	119.6	H1A—N1—H1D	116.2
C4—C3—H3A	119.6	H1B—N1—H1D	108.7
O2—C8—O3	126.13 (10)	H1C—N1—H1D	107.5

### Hydrogen-bond geometry (Å, °)

**Table:**

*D*—H···*A*	*D*—H	H···*A*	*D*···*A*	*D*—H···*A*
O1W—H1···O2^i^	0.85	1.97	3.2969 (15)	169.9
N1—H1A···O2^i^	0.84	2.07	2.8908 (14)	168
N1—H1B···O3^ii^	0.85	2.02	2.8578 (13)	168
N1—H1C···O3^iii^	0.88	2.09	2.9310 (15)	161

Symmetry codes: (i) *x*, −*y*, *z*+1/2; (ii) *x*, *y*+1, *z*; (iii) −*x*, −*y*, −*z*.
